# Editorial: Advancement in photonic sensing for abiotic stress management in horticultural and plant nursery sectors

**DOI:** 10.3389/fpls.2026.1862983

**Published:** 2026-05-12

**Authors:** Silvia Traversari, Sonia Cacini, Lorenza Tuccio, María R. Conesa

**Affiliations:** 1 Research Institute on Terrestrial Ecosystems (IRET), National Research Council of Italy (CNR), Pisa, Italy; 2 Consiglio per la ricerca in agricoltura e l’analisi dell’economia agraria (CREA), Centro di ricerca Orticoltura e Florovivaismo, Pescia (PT), Italy; 3 Institute of Applied Physic “Nello Carrara” (IFAC), National Research Council of Italy (CNR), Sesto Fiorentino (FI), Italy; 4 Water and Crop Production Department, Centro de Edafología y Biología Aplicada del Segura-Consejo Superior de Investigaciones Científicas (CEBAS-CSIC), Murcia, Spain

**Keywords:** plant physiology, non-destructive methodology, optical sensors, imaging, Raman spectroscopy, portable tools

Climate change is intensifying abiotic stresses worldwide, with major implications for agricultural productivity. In the horticultural sector, abiotic stresses are the primary drivers of yield losses, reaching up to 70%. These stresses, including among others drought, salinity, waterlogging, and extreme temperatures, play a crucial role in impacting agronomic management practices (Fraire-Velázquez and Balderas-Hernández, 2013; Gull et al., 2019; Francini and Sebastiani, 2019).

The horticultural and plant nursery sectors encompass highly diverse production systems, ranging from open-field cultivation to protected environments, such as greenhouses and soilless systems. In this context, abiotic stresses compromise crop quality and ornamental value (Hayat et al., 2023), increasing the need for sustainable solutions for growers.

Agronomic practices are evolving towards approaches that enhance resilience while minimizing environmental impacts. Plant breeding, the use of bioactive compounds and beneficial microorganisms, and advances in plant physiology are contributing to mitigating abiotic stress. Moreover, precision agriculture is playing an increasingly relevant role through the real-time monitoring of environmental conditions and plant health, allowing prompt interventions to protect crops.

Despite the growing availability of decision support systems, their adoption by growers is often constrained by high costs and insufficient knowledge transfer. In this sense, further research and field-scale studies are essential to enhance the practical application of these technologies.

Photonic sensing is transforming how plant stress is monitored and managed in horticulture and nursery systems, enabling precision practices that optimize resource use and enhance crop productivity. As technology advances and becomes more affordable, its role in precision agriculture is increasingly relevant.

Leaf−clip optical sensors such as SPAD and Dualex, as well as portable spectrometers and fluorometers, gas−exchange devices, and LAI meters, have long provided rapid assessment of leaf physiology by measuring foliar metabolites, photosynthetic efficiency, and canopy structure, respectively. These tools support the diagnosis of nutrient deficiencies, water stress, and photoinhibition in operational production settings. Recent advances in photonic technologies are expanding these capabilities. Optical Coherence Tomography (OCT) allows the internal tissue imaging, revealing early stress−induced anatomical changes in plants. Portable Raman spectroscopy allows non−destructive detection of biochemical markers (i.e., pigments and stress−related compounds). In parallel, multispectral and hyperspectral imaging combined with 3D techniques enhance whole−plant phenotyping. Integrated RGB and thermal imaging provides spatial information on chlorophyll distribution, water−use efficiency, temperature, and canopy architecture. These approaches bridge the gap between point−based measurements and crop−scale monitoring, enabling continuous plant stress assessment.

The integration of established sensors with cutting−edge photonic imaging provides a robust framework for early stress detection and precision management. This Research Topic gathers 8 Original Research papers exploring plant adaptive responses using several photonic technologies, highlighting the value of combining proximal and remote approaches with advanced analytics to improve resource−use efficiency and crop performance under stress.

The works of Incesu et al. and Dieleman et al. examined the effects of salt stress and light fluctuations, respectively, using SPAD-based chlorophyll and fluorescence measurements. Incesu et al. highlighted the positive role of arbuscular mycorrhizal fungi in enhancing salt stress resistance in persimmon rootstock, while Dieleman et al. showed that extreme light fluctuations reduced biomass, morphology, and chlorophyll content in tomato compared with constant light. By contrast, short-term medium-amplitude fluctuations had negligible effects, indicating that, when minimum light levels are maintained, plants can tolerate dynamic light regimes without impaired development. These findings suggest that light dynamics can be managed as a functional component of cultivation systems rather than solely as a stress factor.

Novel OCT techniques enable micrometer-scale imaging of living tissues (Bouma et al., 2022). Chauvet and Matcher used OCT to study long-range plant signaling, visualizing real-time morphological changes in tomato leaves after wounding. By combining near-infrared imaging with subpixel registration, the authors achieved submicrometric tracking of wound-induced leaf movements, revealing dynamics associated with slow wave potentials and hydraulic signaling. As OCT captures mechanical deformations without physical contact, this approach opens new possibilities for studying real-time plant responses to stress.

Tuccio et al. proposed an integrated multi-sensor optical approach combining Raman sensing with Dualex and Multiplex fluorescence meters to optimize nitrogen fertilization in broccoli. While fluorescence sensors provided indices of pigments and nitrogen balance, portable Raman spectroscopy enabled molecular-level detection of key compounds (i.e*.*, chlorophylls, carotenoids, polyphenols, etc.). Strong correlations with tissue nitrogen content highlighted the value of integrating these techniques for improving nutrient stress assessment.

Imaging approaches were explored by Jeong et al., Liao and Zhang, and Li et al. using different spectral resolutions in combination with other different techniques. Jeong et al., combining RGB imaging, thermal imaging, and chlorophyll fluorescence, achieved an early detection of drought stress in Japanese larch seedlings. Changes in needle angles, measured from RGB images, preceded chlorophyll fluorescence or thermal responses, proving a more sensitive indicator of drought stress than conventional physiological measurements. Liao and Zhang combined multispectral imaging with high-resolution 3D geometry to improve vein segmentation and quantify vein length per unit leaf area, enabling non-destructive vascular analysis where 2D methods often fail. Li et al. integrated photosynthetic traits with leaf hyperspectral reflectance to early detect water stress in buckthorn. Changes in leaf chlorophylls and chlorophyll fluorescence occurred before the appearance of visible stress symptoms. Specific spectral regions and vegetation indices were closely linked to these physiological changes, allowing drought effects to be tracked using hyperspectral data (Ramírez-Cuesta et al., 2022). Machine learning models further enabled accurate estimation of key photosynthetic parameters (Shah et al., 2019), supporting large-scale drought monitoring.

Finally, Bulgari et al. used a leaf-clip spectroradiometer to monitor UV–VIS–NIR leaf reflectance on lettuce grown in a greenhouse soilless system. Vegetation indices related to pigment content allowed an early discrimination of water stress, nutrient deficiencies, and fungal outbreaks before visible symptoms or SPAD changes occurred, highlighting reflectance-based sensing as an effective early-diagnosis tool on leafy vegetables.
